# The Architecture of the Cytoplasmic Region of Type III Secretion Systems

**DOI:** 10.1038/srep33341

**Published:** 2016-09-30

**Authors:** Fumiaki Makino, Dakang Shen, Naoko Kajimura, Akihiro Kawamoto, Panayiota Pissaridou, Henry Oswin, Maria Pain, Isabel Murillo, Keiichi Namba, Ariel J. Blocker

**Affiliations:** 1 Schools of Cellular & Molecular Medicine and Biochemistry, Faculty of Biomedical Sciences, University of Bristol, University Walk, United Kingdom; 2 Graduate School of Frontier Biosciences, Osaka University, Japan

## Abstract

Type III secretion systems (T3SSs) are essential devices in the virulence of many Gram-negative bacterial pathogens. They mediate injection of protein effectors of virulence from bacteria into eukaryotic host cells to manipulate them during infection. T3SSs involved in virulence (vT3SSs) are evolutionarily related to bacterial flagellar protein export apparatuses (fT3SSs), which are essential for flagellar assembly and cell motility. The structure of the external and transmembrane parts of both fT3SS and vT3SS is increasingly well-defined. However, the arrangement of their cytoplasmic and inner membrane export apparatuses is much less clear. Here we compare the architecture of the cytoplasmic regions of the vT3SSs of *Shigella flexneri* and the vT3SS and fT3SS of *Salmonella enterica* serovar Typhimurium at ~5 and ~4 nm resolution using electron cryotomography and subtomogram averaging. We show that the cytoplasmic regions of vT3SSs display conserved six-fold symmetric features including pods, linkers and an ATPase complex, while fT3SSs probably only display six-fold symmetry in their ATPase region. We also identify other morphological differences between vT3SSs and fT3SSs, such as relative disposition of their inner membrane-attached export platform, C-ring/pods and ATPase complex. Finally, using classification, we find that both types of apparatuses can loose elements of their cytoplasmic region, which may therefore be dynamic.

Type III secretion systems (T3SSs) are essential devices in the virulence of many Gram-negative bacterial pathogens. They mediate injection of protein effectors of virulence from bacteria into eukaryotic host cells to manipulate them during infection. T3SSs involved in virulence (vT3SSs) are evolutionarily related to bacterial flagella assembly apparatuses (fT3SSs), which allow cell motility. Both types of macromolecular machines are composed of three major parts: a hollow extracellular portion (needle or flagellum), a basal body -known as the “needle complex” (NC) base for vT3SSs or hook-basal body (HBB) for fT3SSs- spanning both bacterial membranes and containing a membrane-inserted protein export apparatus, and a cytoplasmic export complex. The entire apparatus serves as a conduit for secretion of virulence proteins or flagellar filament components.

The structure of the external and transmembrane parts of both fT3SS and vT3SS is increasingly well-defined^1^, with the identity and location of the majority of the outer and inner membrane components being well-established. However, the arrangement of their cytoplasmic and inner membrane export apparatuses (CIMEA) is much less clear. The cytoplasmic region of one of the five inner membrane export apparatus (IMEA) components, MxiA of *Shigella flexneri* vT3SS and FlhA of *Salmonella enterica* serovar Typhimurium fT3SS, is required to form a nonameric toroid immediately underneath the basal body^2^. Within the cytoplasmic export apparatus (CEA), the cytoplasmic F1-like export ATPase Spa47/FliI forms a homohexameric complex positioned ~5 nm underneath the MxiA/FlhA toroid and 10 to 15 nm down from the membrane-embedded portion of the IMEA^3^. In fT3SSs, FliM and FliN form a large and robust ring surrounding the cytoplasmic face of the basal body, known as the C-ring. Recent electron cryotomography (ECT) studies in *Shigella flexneri* identified densities for six cytoplasmic pods surrounding the base of its vT3SS with the sole FliM-FliN homolog Spa33 being required for their presence and that of the ATPase complex. MxiN, a homolog the dimeric flagellar ATPase inhibitor FliH was only required for the presence of the ATPase complex and was hence postulated to form six additional “linkers” seen to connect the ATPase complex to the pods^4^ (Fig. 1d). Whether such an arrangement is also found in other vT3SSs is unknown. Furthermore, despite the conservation within this protein family, whether FliH forms similar links to the C-ring in fT3SS^5^, where the rotational symmetry of the C-ring shows variable symmetries from 32 to 36^6^, is not yet clear.

Two other CEA components are known, but have not been localised within the cytoplasmic structures so far described via ECT. These are FliJ/Spa13, a homolog of the F1-ATPase γ-subunit, which binds the fT3SS ATPase homohexamer’s central cavity *in vitro*^7^, and MxiK, which interacts with the ATPase^8^ but lacks a clear fT3SS homolog. All components are essential for high efficiency type III secretion. The organisation of the other components within the structures described by ECT is increasingly clear, especially in the better-studied fT3SS. The smaller FliN is similar to the C-terminus of the larger FliM, each being composed of one sequence-related SPOA domain, known as SPOA2 and SPOA1, respectively^9^,^10^,^11^. Most vT3SS systems encode a single protein in place of FliM and FliN. This protein is as large as FliM and resembles FliM at its N-terminus but is more like FliN at its C-terminal domain where it carries both a SPOA1 and a SPOA2 domain. However, in *Yersinia*, *Salmonella* and *Shigella* vT3SSs, smaller proteins that are similarly sized to FliN and contain just the SPOA2 domain are generated from the same genes through unconserved internal translational initiation start sites, and they are essential for function^12^,^13^,^14^. Recently, it was shown that the smaller protein homodimerises and forms a heterotrimeric complex with the C-terminal SPOA2 domain of the larger one^11^,^14^. Thus, this complex contains four SPAO domains. The ratio of FliM to FliN in the flagellar C-ring *in situ* was estimated as 1:3^15^, and this was confirmed recently *in vitro*^14^. Because FliM contains only a single SPAO domain, the number of SPAO domains in the FliM-FliN complex is also four. This implies that the fT3SS C-ring is structurally equivalent with the vT3SSs counterpart.

However, what remains unclear is whether and how these structurally similar complexes form rings in fT3SSs and pods in vT3SSs. The full length *Yersinia* vT3SS Spa33 homolog was found in 22 + /−7 to 8 copies per basal body. This suggests there is one copy of the SPAO domain heteroteramer per pod. However, the full composition of the pod and its mode of binding to the vT3SS inner membrane-spanning MxiG ring with 24-fold rotational symmetry remain unclear. Furthermore the *Shigella* Spa33 full length (Spa33-FL) and its C-terminal fragment (Spa33-C) associate as Spa33-FL/C_2_ that further oligomerises into elongated arrays *in vitro*, which can be manually positioned in the EM density corresponding to the cytoplasmic edge of the *Salmonella* Typhimurium fT3SS C-ring with the 34-fold rotational symmetry^6^,^14^. This lead to questioning of whether the six pods seen might just represent an incomplete view of a dynamic vT3SSs C-ring^14^. Indeed, full-length YscQ from *Yersinia*, which is a homolog of Spa33, was shown to be dynamic, with a turnover half-time of about 2 min, which was reduced by an active export ATPase^16^. This finding, the considerable physical distance separating the export ATPase from the export platform and the uncertainty over the route that exported proteins take across the system, also suggest several states of the export apparatus might be discernable.

Here we investigate using ECT the structures of the cytoplasmic regions of the vT3SSs of *Shigella flexneri* and *Salmonella enterica* serovar Typhimurium, where the latter is also known as SPI1, and that of the fT3SS *Salmonella* Typhimurium and compare them to determine the common and different structural features. We show that the cytoplasmic regions of vT3SSs display conserved six-fold symmetric features including pods, linkers and an ATPase complex, whereas fT3SSs probably display the six-fold symmetry only in their ATPase region. In addition, we identify morphological differences between vT3SSs and fT3SSs, such as relative disposition of their inner membrane-attached export platform, C-ring/pods and ATPase complex. Finally, using classification, we find that both types of apparatuses can loose elements of their cytoplasmic region, which may therefore be dynamic.

## Results and Discussion

### The cytoplasmic region of the *Shigella flexneri* vT3SS displays C6 symmetry

**Figure 1:**
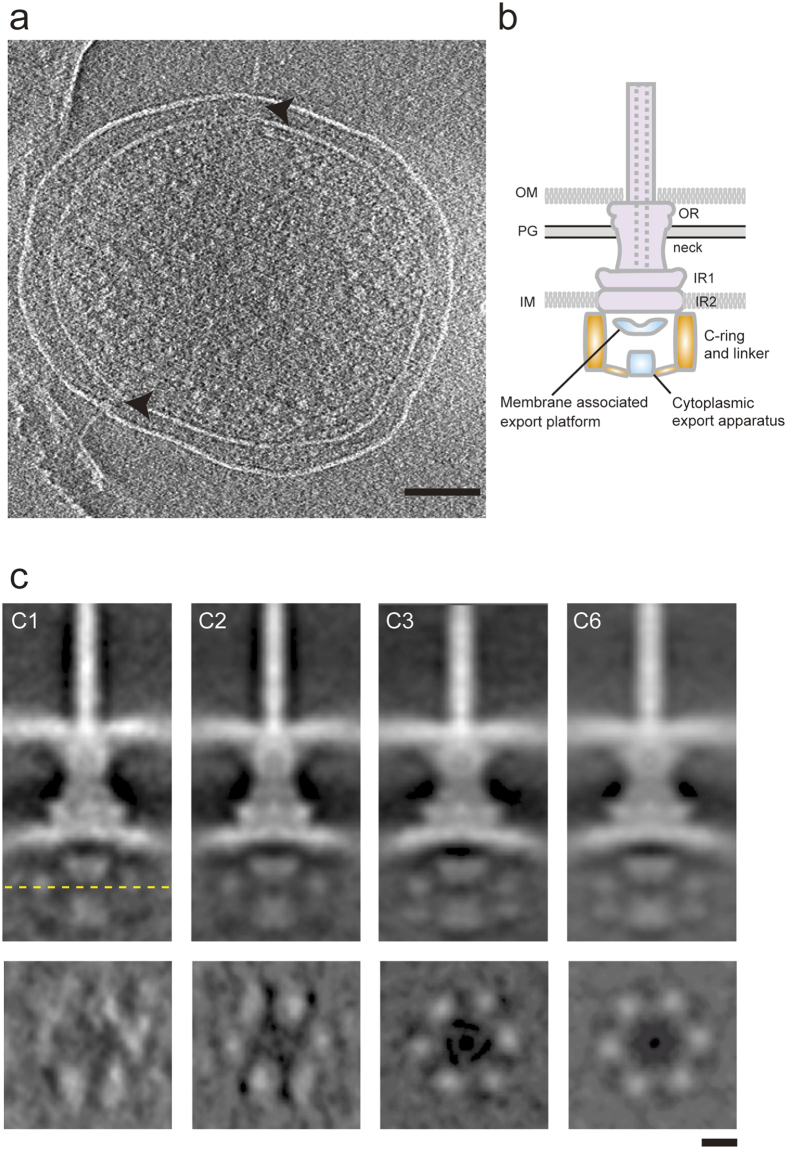
ECT of *Shigella flexneri* minicell and subtomogram averaging maps of its Needle Complex with various symmetries. (**a**) Central slice of a tomographic reconstruction of a typical minicell showing two needle complexes embedded in the cell membranes. Scale bar indicates 100 nm. (**b**) Schematic diagram of a NC embedded in the cell membrane. *Purple* color indicates *in vitro* structures from isolated NC, *blue* color indicates known position of a component of the inner membrane export apparatus (export platform) and *orange* color indicates cytoplasmic export apparatus (CEA), as defined by a new study^4^: ATPase, pods and linkers. Abbreviations are as follows: inner membrane, IM; outer membrane, OM; peptidoglycan layer, PG; outer region, OR; inner regions, IR1 and IR2; IMR inner membrane region; OMR, outer membrane region. (**c**) *Top,* central sections of subtomogram averaging maps of NCs with C1, C2, C3 and C6 symmetry, respectively, and *below* each corresponding cross sections at height indicated by the dashed yellow line. The cross section of map with C1, C2, C3 and C6 symmetry shows six “blobs”, corresponding to the pod-like regions. Scale bars indicate 10 nm.

We generated minicells by over expression of *Salmonella* FtsZ in *Shigella flexneri*. We confirmed the presence of the needle complexes in the minicells (Fig. 1a), as found in previous reports for *Yersinia*, *Salmonella* and *Shigella* spp.^4^,^17^,^18^. Recently, Hu *et al.* reported six “pod” densities, corresponding to the C-ring structure in HBBs, in the cytoplasmic region of the *Shigella flexneri* NC visualized *in situ*. This included also six linker densities joining the ATPase and the pods (Fig. 1b). This had never been visualized in NCs, even from other species. We picked 265 subtomogram particles, manually aligned them by eye, and used Dynamo^19^ for subtomogram averaging. We performed CTF-correction using TOMOCTF. When we applied C50 symmetry, we, like others^17^,^18^, could not detect densities of the pods and linkers. However, when we applied C6 symmetry in our procedure we could detect six pod densities and six linkers (Fig. 1c), very similar in dimensions (width of cytoplasmic region 32 nm and height 20 nm between top of MxiAc and bottom of Spa47), and shape, to those identified by Hu *et al.*^4^ in the cytoplasmic region of the *Shigella* vT3SS. Furthermore, when we generated three-dimensional (3D) averages without imposing symmetry (C1; Fig. 1c) or by imposing two and three-fold rotational symmetry (C2 and C3; Fig. 1c), we also detected six pod densities in the cytoplasmic region. When we applied C1, C2 and C3 symmetry, we used the volume resulting from a 3D-reconstruction applying C101 symmetry as a reference volume to avoid reference bias. The fact that the six pods are visible no matter what symmetry and starting model strongly supports their existence *in situ*. How the six pods are associated at regular intervals with the cytoplasmic face of MxiG inner membrane ring, which displays C24 symmetry^20^, remains to be determined. There must be a specific structural arrangement for Spa33 heterotrimers binding to a group of four MxiG subunits to build up an architecture with such a symmetry mismatch.

### Structural variation within the cytoplasmic region of the *Shigella flexneri* vT3SS

**Figure 2:**
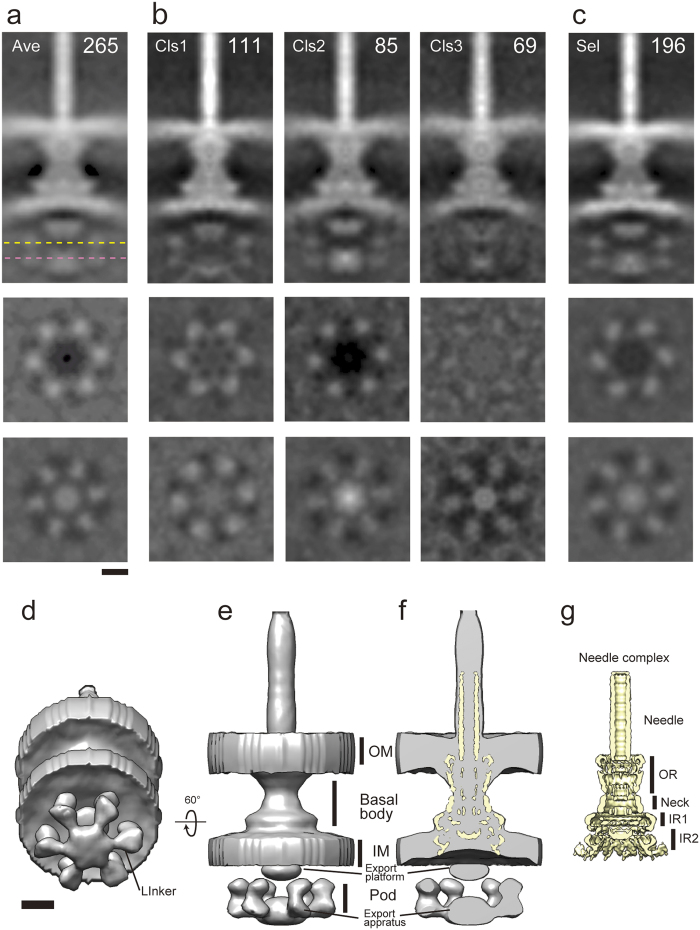
Classification of the Needle Complex subtomogram averages from *Shigella flexneri* and superimposition of *in vitro* map on *in situ* map. (**a–c**) Central/cross section maps of original subtomogram averaging map of the NC (**a**) and three classes (**b**), *middle* and *bottom* each corresponding cross sections at height indicated by the dashed yellow line and the dashed pink line, respectively. The number of particles in each class is indicated in the *top right corner*. (**c**) Refined map excluding class 3, which shows weak densities in cytoplasmic region. (**d,e**) The 3D rendering maps show pod-like densities, linker densities between membrane associated export apparatus (export apparatus) and pods, export apparatus and cytoplasmic export platform (export platform). (**f,g**) Superposition of *in vitro* NC map obtained from single particle image analysis (**g**, *yellow*) on *in situ* map from subtomogram averaging (**f**, *grey*). *In situ* and *in vitro* NC map show OR, Neck, IR1 and IR2. Scale bar indicates 10 nm.

Since a vT3SS cytoplasmic region protein Spa33 homolog displays dynamic behavior in *Yersinia* as monitored by super-resolution fluorescence microscopy^16^, we investigated morphological variation within the NC cytoplasmic region. For this we used classification by multireference alignment within the cytoplasmic region only, having masked out the rest of the structure, using the subtomogram average obtained during 3D-averaging with C6 symmetry (Fig. 2a). The shape of the inner membrane profile, the appearance of the MxiA_C_ toroid and the height of the ATPase complex differ between classes (Fig. 2b). We also noticed that the densities of ATPase in class 1 and that of the pods and ATPase in class 3 were weaker than those in class 1. The degree and size of inter-class variation suggests that the cytoplasmic region of vT3SSs undergoes large-scale conformational changes.

### Comparison between *in situ* and *in vitro* NC structure

As the cytoplasmic region did not appear clearly in class 3, we refined the subtomogram averaging excluding class 3 (Fig. 2b). The cytoplasmic region became clearer than before in the new 3D map (Fig. 2c–e). We used this map for comparison with the density map of the NC obtained from single particle image analysis of cryoEM data (Fig. 2g; Kajimura, Cheung, Blocker and Namba, unpublished). The periplasmic regions of both maps are morphologically in good agreement (Fig. 2f). Liu’s group proposed that the MxiG cytoplasmic region protrudes into the pod region^4^. We see similar features in both tomograms and single particle image reconstructions. As previously reported^17^,^18^, the NC volume obtained by single particle reconstruction is ~5 nm shorter in its outer membrane region than the *in situ* NC volume obtained from ECT data. The map from Liu’s group shows more detail in the outer membrane region (OMR), which extends into the OR (Fig. 1b) of NC associated elements, and this part may be formed by the pilotin MxiM^21^,^22^.

### Co-instability of *Shigella* vT3SS CEA components

Analysis of differences between ECT maps from WT and deletion mutants as a means to assign the location of the missing components^4^ relies on the assumption that in the absence of one, the others are stable, and then correctly localized. We tested this first assumption for all CEA proteins, mainly using FLAG-tagged constructs as we failed to generate antibodies against most of these proteins that detect them at native levels in whole bacteria. Evidently, co-stabilisation does not equate to incorporation into the same multi-protein complex(es). However, we previously reported that His6-Spa47, when expressed in Δ*spa47* at its native level, co-purifies in complex with Spa33, MxiN and MxiK^23^.

**Figure 3:**
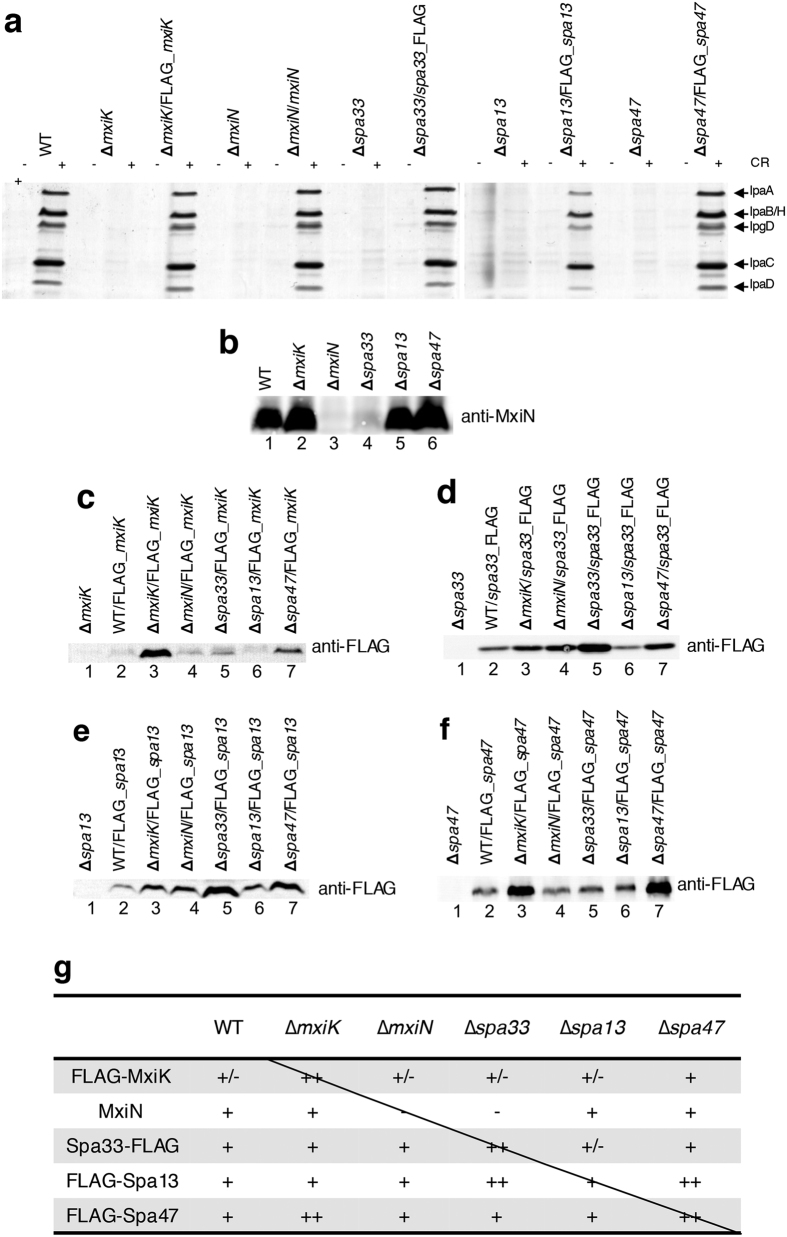
Analysis of the stability or expression of components of the *Shigella* T3SS cytoplasmic export apparatus in mutants of this portion of the apparatus. (**a**) Complementation of cytoplasmic export apparatus knock mutants by *in-trans* expression of wild-type or FLAG-tagged proteins. Induced secretion of Ipa proteins in the presence (+) or absence (−) of Congo red (CR) was analysed by silver staining. The wild-type (WT) and the deletion mutants were used as controls, and bacterial numbers were normalized by optical density. The positions of the major Ipa proteins detected by silver staining are indicated on the side. The data shown here are representative of 2 independent experiments giving similar results. (**b–f**) Expression of native MxiN (**b**) and FLAG-tagged MxiK (**c**), Spa33 (**d**), Spa13 (**e**) and Spa47 (**f**) in different mutant backgrounds. Pellets of overnight grown cultures with equivalent bacterial numbers were analysed by immunoblotting with either a polyclonal rabbit antiserum against MxiN (**b**) or a monoclonal antibody against the FLAG tag (**c–f**), as indicated at the side. The data shown here are representative of 2 independent experiments giving similar results. The blots in b-f were cropped to show only the area where signal was detected. (**g**) Table semi-quantitatively summarising results of co-stability experiments in (**b–f**).

Firstly, we checked the activity of *Shigella* vT3SSs from deletion mutants, using Congo red (CR) as a selective, artificial inducer of secretion in this species (Fig. 3a 24). In the Δ*mxiK*, Δ*mxiN*, Δ*spa33*, Δ*spa13* and Δ*spa47* mutants, no secretion of early effector proteins (IpaA, IpaB, IpaC, IpaD and IpgD) was observed, as previously reported, due to lack of secretion of any components of the apparatus beyond the inner membrane^8^,^25^,^26^. We also confirmed restoration of inducible secretion activity of vT3SSs from these deletion mutant strains complemented with the corresponding constructs encoding wild-type (*mxiN*) or FLAG-tagged proteins (Fig. 3a). This indicates all mutants are non-polar on downstream *mxi* genes and the FLAG-tagged constructs are functional.

Next, we checked the stability of each of the native or FLAG-tagged components in the WT strain and deletion mutants of the others five components (Fig. 3b–g). MxiN, for which we have a quality antibody, was detected in all mutants, except in Δ*spa33*, where it was completely absent (Fig. 3b). This indicates MxiN requires Spa33 for stability and a Δ*spa33* mutant is phenotypically identical to a Δ*spa33* Δ*mxiN* double mutant. In mutants of all the other FLAG-tagged components expressed, the FLAG-tagged proteins were more abundant than in the WT strain (Fig. 3: panel c, lanes 2 and 3; panel d, lanes 2 and 5; panel e, lanes 2 and 6; panel f, lanes 2 and 7). This suggests there is competition for CEA complex formation between the WT proteins and their tagged equivalents in the WT background and that excess, uncomplexed, tagged molecules are unstable. Whilst can assume from Fig. 3a that the FLAG-tagged proteins are equally functional to the WT ones and hence compete equally well with them, we have no way of estimating what each of their relative expression ratios are. But, we can assume, since the expression of all FLAG tagged proteins is from the *lac* promoter in pUC19, that each individual tagged protein is expressed at the same level in each strain. Therefore the fact that FLAG-MxiK is detected inΔ*mxiK* but not in WT (Fig. 3c, lanes 2 and 3), suggests that the stability of FLAG-MxiK is enhanced in a background which does not express WT MxiK. FLAG-MxiK is also stabilized, to a lesser extent, in Δ*spa47* but not in the other deletion mutants (Fig. 3c, lane 7). Taken together with the first observation, this suggests WT MxiK is partially destabilised in Δ*spa47* but not in the other mutants. Together these data suggest that native MxiK requires Spa47 for stability. This is supported by the fact that FLAG-Spa47 is more stable in Δ*spa47* and Δ*mxiK* than in WT or any of the other mutants (Fig. 3f, lanes 3 and 7). This also suggests, conversely, that Spa47 requires MxiK for stability.

Spa33_FLAG is less stable in WT and Δ*spa13* than in all the other mutants (Fig. 3d, lanes 2 and 6). This conversely suggests native Spa33 requires MxiK, MxiN and Spa47 for stability. Using the same reasoning, Spa13 seems to require primarily Spa47 and Spa33 for stability, but also perhaps MxiK and MxiN (Fig. 3e). To summarize (Fig. 3g), in the Δ*spa33* mutant examined by Hu *et al.*^4^, in which the entire NC cytoplasmic region was lacking, it is likely that MxiN and Spa13 levels were substantially reduced, and that, as a consequence, any expressed Spa47 was mislocalized. In the Δ*mxiN* mutant Hu *et al.* examined, MxiK, Spa33 and Spa47 would have been present but Spa13 was likely unstable and the ATPase complex hence mislocalized. This explains why not only the linker regions, probably formed of MxiN, but also the ATPase portion is lacking in their ECT map. A Spa13 monomer is too small and/or dynamic to be localised by ECT at currently achievable resolutions and deletion of *mxiK* has pleiotrophic effects, most notably on Spa47 stability, which itself might affect the localization of other CEA components. Therefore, we chose not to attempt to localise these components using difference maps here. However, our past co-precipitation data^23^, and now co-stability data, suggest they are closely associated with Spa47.

### Subtomogram averaging in the *Salmonella* vT3SS with C6 symmetry

To determine whether the six-fold symmetry of the CEA was found also in other vT3SSs, we re-picked 347 NC subtomogram particles from the *Salmonella* minicell ECT data, with which Kawamoto *et al.*^17^ have already published a structure obtained by subtomogram averaging with C100 (cylindrical) symmetry and without CTF correction. We then performed CTF correction using TOMOCTF and subtomogram averaging using Dynamo as for the *Shigella* vT3SS, with C6, C1, C2 and C3 symmetry imposed.

**Figure 4:**
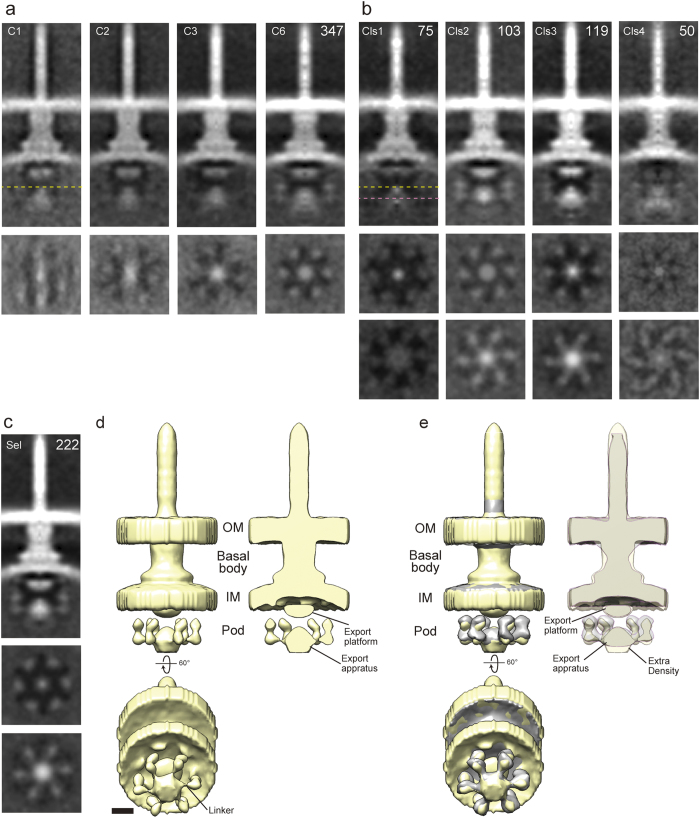
ECT subtomogram averaging map of *Salmonella* Typhimurium. **(a)** central sections from the subtomogram averaging map of the NC in *Salmonella* with C1, C2, C3 and C6 symmetries, respectively and *below* each corresponding cross sections at height indicated by the dashed yellow line. **(b)** Central and cross section of four classes and *middle/bottom* each corresponding cross sections at height indicated by the dashed yellow line and the dashed pink line, respectively. The number of particles for each class is indicated in the *top right corner*. **(c)** Refined map excluding class 1 and 4, which show only weak densities in cytoplasmic region. **(d)** 3D surface rendering from *in situ* map of *Salmonella* showing NC and cytoplasmic regions. *Below,* the map inclined 60° to show the export apparatus, including extra density and linkers. *Top right,* a central section of the map. **(e)** Comparison of *in situ* maps of *Shigella flexneri (grey*) and *Salmonella* Typhimurium T3SSs (*yellow*). *Below,* the maps inclined 60° to show the export apparatus and linkers. *Top right,* overlay of central sections of these maps. Scale bar indicates 10 nm.

We confirmed the six-fold rotational symmetry of the *Salmonella* vT3SS cytoplasmic region, which we had not observed so far, even in the maps with C2 and C3 symmetry imposed (Fig. 4a). It is likely that the structure of the C-ring region was smeared in the previous study by the C100 symmetry applied^17^ to smooth the cylindrical features of the basal body. Next, we performed 3D classification by multireference alignment using Dynamo. Several of the classes showed variation in their inner membrane and InvA_C_ regions (Fig. 4b). We removed class 1 and 4 (Fig. 4b), which seemed to lack the pods, for refinement. The result showed six linkers and pods (Fig. 4c,d). In addition, class 3 and the refined volume showed extra density under the ATPase, not seen in the *Shigella* vT3SS structure. The difference is shown in Fig. 4e by direct comparison of these two structures by superposition. The extra density may represent hexameric chaperone/effector complexes docked to the ATPase complex prior to substrate export, as proposed for SopB/SigE in *Salmonella* by Roblin *et al.*^27^.

### Subtomogram averaging of the Salmonella fT3SS with C6 symmetry

FliH is a homolog of the MxiN linker protein, forms dimers and binds one FliI ATPase molecule^28^ to build a FliH_12_FliI_6_FliJ ring below the FlhA_C_ toroid^29^. This indicates that two FliH molecules make one linker and suggests they extend to the C-ring region, like in vT3SSs. Therefore, we also performed subtomogram averaging with C6 symmetry on ECT data from the *Salmonella* fT3SS^17^ to test for the appearance of six linker densities between the ATPase and C-ring. But, no linker densities could be observed in the cytoplasmic region below the HBB (Supplementary Figure S3a, right). Interestingly, classification of these particles also reveals a class (class 3) lacking clear features for the C-ring and ATPase complex and one (class 1), lacking a clear ATPase complex.

### Conclusions from comparison of the cytoplasmic region of different T3SSs

As a result of subtomogram averaging with C6 symmetry, we respectively confirmed and revealed six pods, corresponding to the fT3SS C-ring, and six linker densities in the cytoplasmic regions of the vT3SSs of *Shigella* and *Salmonella* species. However, we could not observe linker densities in the flagellar HBB. The fT3SS C-ring displays 32 to 36-fold symmetry although there are constantly only six linkers given the hexameric nature of the ATPase. Given that these linkers are readily seen in the vT3SS maps, which are at similar resolution to the HBB one, the fT3SS linkers may be more flexible and hence able to interact dynamically with several nearby C-ring subunits, leading them to become smeared out by the imposition of C6 symmetry.

**Figure 5:**
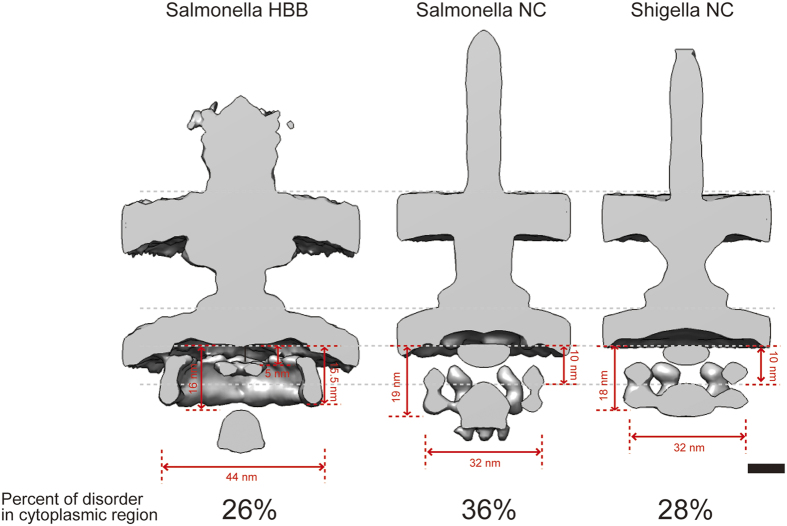
Comparison between fT3SS and vT3SSs. Centrally sectioned maps of *Salmonella* HBB, NC and *Shigella* NC. Gray dotted lines indicate the position of, from top, outer and inner membranes, export platform, export apparatus. Red numbers indicate distances between export platform and apparatus, width of the pod/C-ring region and size of the ATPase region. At the bottom, numbers show percent of particles of each kind entirely lacking features in their cytoplasmic region.

Parts of the T3SS export apparatuses have common features and function. However, the structure of those parts can display different morphologies (Fig. 5). In particular, the position of FlhA_C_/MxiA_C_/InvA_C_ toroid, also termed the export platform, differs by ~5 nm between fT3SS and vT3SS. The position of the ATPase also differs between them, being ~6 nm further below the export platform in fT3SSs. Furthermore, the fT3SS C-ring is ~12 nm wider and ~4 nm shorter than the vT3SS pods. We presume the fT3SS C-ring is positioned closer to the cytoplasmic membrane in order for its top portion to interact with the MotA-MotB stator complex unit located therein for motor torque generation^30^. This function is not found in vT3SS.

The ATPase is supported into its position by the FliH/MxiN family of linkers. Therefore, Abrusci *et al.* proposed that the position of the ATPase is controlled by the length of the FliH/MxiN family proteins, as well as by the height and width of the C-ring/pods^2^. However, despite quite different relative positions of the ATPase complex in their species of origin, FliH and MxiN are both ~230 residues long. This suggests that additional factors control the morphological differences we observe. For instance, FliJ, the γ-subunit equivalent in *Salmonella* flagella, is 147 residues long when its *Shigella* vT3SS homolog Spa13 is only 112 residues long. This length difference would lead ~53 Å reduction in length in vT3SS homologs, assuming 1.5 Å per residue of α-helix, reduced maximally to ~26 Å, given that these proteins partially fold upon themselves to form intramolecular α-helical coiled coils^7^. As FliJ inserts into the central cavity of the FliI ATPase hexamer and binds FlhA^7^,^31^, it may connect the CEA to the FlhA export platform, perhaps explaining the ~30 Å height difference in position we see between fT3SSs and vT3SSs ATPase complexes.

Between the vT3SSs we analysed, the main difference is the extra density under the ATPase region of the *Salmonella* vT3SS, which may be formed by hexameric chaperone-substrate complexes^27^. Indeed, the vT3SS of *Salmonella* shows greater secretion activity under the growth conditions we used^32^ than that of *Shigella*, which requires induction. However, from our data, we cannot determine which map best reflects the state of active export. For this, we need to analyse in parallel mutants locked in specific secretion modes^33^, something we have tried but failed to obtain quality data for so far.

Finally, upon classification, we revealed that 26~36% particles of fT3SS and vT3SSs lack clear CEA features. Therefore, not all T3SSs have C-ring/pod and ATPase regions, and these are found at similar ratios for each T3SS studied. Interestingly, this is also observed in *Borrelia* flagellum, where a similar fraction appeared to lack part of the C-ring^34^. One possibility is that these particles have lost their C-ring/pods and ATPase structure because they are damaged by the preparation of minicells or in a non-active state for other artefactual reasons. However, vT3SSs are known to have dynamic components in their cytoplasmic domains^16^. Therefore, this finding could also support the notion that the entire CEA is dynamic in most T3SSs. This notion is strengthened by recent study by ECT and sub-tomogram averaging to examine the structure of the *Chlamydia trachomatis* T3SS in the presence and absence of host membrane contact. Comparison of the averaged structures indicated that a ~4nm compaction of the basal body occurs when the needle tip contacts the host cell membrane. This compaction was coupled to a stabilization of the cytoplasmic export apparatus^35^. We did not see an overall compaction in classes containing the ATPase and associated complexes. But, given that 2/3 less particles were collected for the host cell contact condition, the enhancement of the signal seen from the cytoplasmic parts of the T3SS during host cell contact may correspond to an increased number of T3SS possessing these parts when they are likely to be performing effector transport.

## Methods

### Bacterial strains and cell culture

All bacterial strains and plasmids used in this study are listed in Supplementary Table S1. *S. flexneri* strains were maintained and selected on CR agar plates^36^ and grown at 37 °C in trypticase soy broth (TCSB; Becton Dickinson) supplemented with antibiotics when necessary (100 μg of ampicillin ml^−1^, 50 μg of kanamycin ml^−1^, 20 μg of chloramphenicol ml^−1^; Sigma).

### Molecular cloning

All primers used in this study are listed in Supplementary Table S2 and all constructs were verified by DNA sequencing (Eurofins). Constructs encoding N-terminally FLAG-tagged MxiK, Spa13, Spa33 and Spa47 were obtained via PCR using primers listed in Supplementary Table S2 and ligated into pUC19 via *Xba*I/*BamH*I. The C-terminally FLAG-tagged Spa33-encoding construct was also made as an N-terminally FLAG-tagged Spa33-encoding construct but failed to complement Δ*spa33*.

### Analysis of Ipas secretion and protein expression

The secretion of Ipa proteins after Congo red induction was carried our as previously described^37^. The total level of protein expression was revealed via western blot using either monoclonal anti-FLAG (Sigma) or polyclonal anti-MxiN^23^. Goat anti-mouse DyLight 800 (Fisher Scientific) or goat anti-rabbit Alexa 680 (Invitrogen) conjugates were used as secondary antibodies. The membranes were then visualized using an Odyssey infrared imaging system (LI-COR Biosciences).

### Preparation of minicells

We transformed WT *Shigella flexneri* M90T serotype 5a with the pBAD24::*ftsZ* plasmid^17^. 1 ml of overnight culture was added to 100 ml of TCSB, including the appropriate antibiotic. The culture was incubated at 35 °C, shaking at 160 rpm until an OD_600_ of ~0.5 was reached. Then, 0.2% v/v arabinose was added. The culture was further incubated at 35 °C shaking at 160 rpm until an OD_600_ of ~1.0 was reached. The culture was centrifuged at 3,000 g for 7 min to remove the large cells. To collect the minicells, the supernatant was centrifuged at 20,000 g for 5 min and the pellet resuspended in 30 μl of TCSB. To supply a gold fiducial maker, 150 μl of 10 nm nanogold (Sigma) were mixed with 50 μl of 5% w/v BSA, centrifuged at 14000 rpm for 15 min, and the supernatant removed. The sample solution was mixed with gold pellet.

### ECT data collection and tomogram reconstruction

We performed ECT data collection as previously described^17^, with some modifications. Quantifoil molybdenum, 200 mesh, R0.6/1.0 micromachined holey carbon grids (Quantifoil Micro Tools) were glow discharged. 2.7 μl of sample solution supplemented with 10 nm diameter of nanogold was applied to the grid, blotted with filter paper, and plunged into liquid ethane using a Vitrobot (Mk2, FEI), setting blot time to 5 seconds, humidity 100% at 4 °C. Images were collected at liquid-nitrogen temperature using a Titan Krios FEG transmission electron microscope (FEI) operated at 300 kV on a Falcon II direct electron detector (FEI). The pixel size was 0.57 nm. Single-axis tilt series were collected covering an angular range from −65° to +65° with a nonlinear Saxton tilt scheme at 5–10 μm underfocus using the Xplore 3D software package (FEI). A total dose of 200 e^−^/A^2^ or less was used for each tilt series. Images were binned two fold and 3D reconstructions were calculated using the IMOD software package^38^. We applied CTF correction to those tilt series as calculated by TOMOCTF^39^. The *Salmonella* minicell data used here were collected in previous work^17^.

### Subtomogram averaging

Individual NCs and HBBs were manually picked up using the IMOD software package with 100 × 100 × 100 pixel box size for *Shigella* NCs, 128 × 128 × 128 pixel box size for *Salmonella* NCs, 128 × 128 × 128 pixel box size for *Salmonella* HBBs. By this procedure, the orientations of particles were visually aligned, so that the needle/hook portion pointed vertically upwards (z-axis) and the base faced downwards, and were recorded in text files to use them as initial parameters in the Dynamo software package (19), which performed subtomgram averaging. These parameters were also used in this package to correct for the missing wedge. The initial volume for subtomogram average was produced manually in Dynamo using a sum of aligned NCs or HBBs with C101 symmetry.

We also prepared a tight mask containing the needle/hook structures, inner, outer membrane, basal body and cytoplasmic region in real space (Supplementary Figure S1a). The tight mask was applied to each particle during each iteration. In the first four iterations, the searching parameters were roughly with 2 times the binning volume (C6: in plane rotation 30 degree, axial rotation 60 degree, 2 degree increments, C1-C3: in plane rotation 30 degree, axial rotation 360 degree, 5 degree increments) and then the searching parameters were refined in a further 8 iterations without binning (in plane rotation 10 degrees, axial rotation 10 degrees, 1-degree increments and maximum allowed searching 2 voxels). During processing, we applied C1, C2, C3 or C6 symmetry. The resolution was estimated by Fourrier Shell Correlation (FSC) using the gold-standard procedure in Dynamo, in which the two independent data sets produced by dividing the particles contributing to the final average into two halves were used in subtomgram averaging independently (Supplementary Figure S2). The 0.143 threshold criterion was used. The resulting 3D maps were rendered using UCSF Chimera.

### Classification of subtomogram averages

Classification by multireference alignment in Dynamo was performed by first generating 3–4 initial references by randomly adding 1–2 sigma Gaussian noise using EMAN^40^ to an average yielded from the final result of subtomgram averaging with C6 symmetry. During the procedure, particles were masked in the cytoplasmic region (Supplementary Figure S1b), and fixed to prevent rotation and translation.

### Single particle cryoEM map of Shigella NC

NCs were affinity-purified from strains with *mxiG*^*−*^/N-His_6_-*mxiG*, using a protocol described previously from Cheung *et al.* Here, Ni-NTA agarose beads incubation was shortened to 2 hours. A 3.0 μl sample was applied onto a Quantifoil holey carbon molybdenum grid (R 0.6/1.0), which was layered with continuous thin carbon to ~10 nm thickness in house, at 4 °C and was plunge-frozen into liquid ethane using a fully automated vitrification device (Vitrobot, FEI). The specimen was observed at temperatures of 80 K using a JEOL JEM3200FSC electron microscope, an Ω-type energy filter, and a field-emission electron gun operated at an accelerating voltage of 200 kV. Micrographs were recorded under low-dose conditions on a CCD camera (TemCam-F415MP, TVIPS) at a magnification of 91,000 and an electron dose of ~40 electrons/Å^2^. Defocus and astigmatism in the images were determined using CTFFIND3. Approximately 8,000 images of NCs were boxed into ~50,000 particle images with side-views of 320 × 320 pixels using a boxer program from EMAN^40^. Those images were then normalized. Image analysis was performed largely with SPIDER^41^ and RELION^42^ packages. After 2D-classification in RELION to select good particles and side-views, we applied C12 symmetry during the three-dimensional reconstruction by using SPIDER package since we previously reported that this is the largest common denominator between OMR and IMR symmetries^20^.

## Additional Information

**Accession Codes**: The three sub-tomogram averages of T3SSs reported in this study have been deposited in the EM databank with accession numbers EMD-8096 (*Shigella* vT3SS), EMD-8122 (*Salmonella* vT3SS), EMD-8121 (*Salmonella* fT3SS).

**How to cite this article**: Makino, F. *et al.* The Architecture of the Cytoplasmic Region of Type III Secretion Systems. *Sci. Rep.* **6**, 33341; doi: 10.1038/srep33341 (2016).

## Supplementary Material

Supplementary Information
