# Supplementary material for: The Architecture of the Cytoplasmic Region of Type III Secretion Systems
Source: Sci Rep. 2016 Sep 30;6:33341. doi: 10.1038/srep33341 (PMC5043178; doi:10.1038/srep33341)
Supplement: Supplementary Information [file srep33341-s1.pdf]

SUPPLEMENTAL MATERIAL

FOR

The Architecture of the Cytoplasmic Region of Type III Secretion Systems

Fumiaki Makino[a,b], Dakang Shen[a], Naoko Kajimura[a,b], Akihiro Kawamoto[b], Panayiota Pissaridou[a], Henry Oswin[a], Maria Pain[a], Isabel Murillo[a], Keiichi Namba[b] and Ariel J. Blocker[a]

Schools of Cellular & Molecular Medicine and Biochemistry, Faculty of Biomedical Sciences, University of Bristol, University Walk, United Kingdom[a]; Graduate School of Frontier Biosciences, Osaka University, Japan[b].

Supplementary Tables S1 & S2
References
Supplementary Figures Legends S1-S3
Supplementary Figures S1-S3

TABLE S1. *Shigella flexneri* strains used in this study

Strain	Genotype (strain; plasmid)	Reference
WT	Wild-type M90T, serotype 5a	(1)
WT for minicells	WT; pBAD24 *Salmonella ftsZ*	This study and (2)
WT/FLAG_*mxiK*	WT; pUC19 *mxiK* with N-terminal FLAG	This study
WT/*spa33*_FLAG	WT; pUC19 *spa33* with C-terminal FLAG	This study
WT/FLAG_*spa13*	WT; pUC19 *spa13* with N-terminal FLAG	This study
WT/FLAG_*spa47*	WT; pUC19 *spa47* with N-terminal FLAG	This study
Δ*mxiK*	WT with deletion of *mxiK*	(3)
Δ*mxiK*/FLAG_*mxiK*	Δ*mxiK*; pUC19 *mxiK* with N-terminal FLAG	This study
Δ*mxiK*/*spa33*_FLAG	Δ*mxiK*; pUC19 *spa33* with C-terminal FLAG	This study
Δ*mxiK*/FLAG_*spa13*	Δ*mxiK*; pUC19 *spa13* with N-terminal FLAG	This study
Δ*mxiK*/FLAG_*spa47*	Δ*mxiK*; pUC19 *spa47* with N-terminal FLAG	This study
Δ*mxiN*	WT with deletion of *mxiN*	(3)
Δ*mxiN*/*mxiN*	Δ*mxiN*; pJN38	(3)
Δ*mxiN*/FLAG_*mxiK*	Δ*mxiN*; pUC19 *mxiK* with N-terminal FLAG	This study
Δ*mxiN*/*spa33*_FLAG	Δ*mxiN*; pUC19 *spa33* with C-terminal FLAG	This study
Δ*mxiN*/FLAG_*spa13*	Δ*mxiN*; pUC19 *spa13* with N-terminal FLAG	This study
Δ*mxiN*/FLAG_*spa47*	Δ*mxiN*; pUC19 *spa47* with N-terminal FLAG	This study
Δ*spa33*	WT serotype 2a with deletion of *spa33*	(4)
Δ*spa33*/FLAG_*mxiK*	Δ*spa33*; pUC19 *mxiK* with N-terminal FLAG	This study
Δ*spa33*/*spa33*_FLAG	Δ*spa33*; pUC19 *spa33* with C-terminal FLAG	This study
Δ*spa33*/FLAG_*spa13*	Δ*spa33*; pUC19 *spa13* with N-terminal FLAG	This study
Δ*spa33*/FLAG_*spa47*	Δ*spa33*; pUC19 *spa47* with N-terminal FLAG	This study
Δ*spa13*	WT with deletion of *spa*13	(5)

Δ*spa13*/FLAG_*mxiK*	Δ*spa13*; pUC19 *mxiK* with N-terminal FLAG	This study
Δ*spa13*/*spa33*_FLAG	Δ*spa13*; pUC19 *spa33* with C-terminal FLAG	This study
Δ*spa13*/FLAG_*spa13*	Δ*spa13*; pUC19 *spa13* with N-terminal FLAG	This study
Δ*spa13*/FLAG_*spa47*	Δ*spa13*; pUC19 *spa47* with N-terminal FLAG	This study
Δ*spa47*	WT with deletion of *spa*47	(3)
Δ*spa47*/FLAG_*mxiK*	Δ*spa47*; pUC19 *mxiK* with N-terminal FLAG	This study
Δ*spa47*/*spa*33_FLAG	Δ*spa47*; pUC19 *spa33* with C-terminal FLAG	This study
Δ*spa47*/FLAG_*spa13*	Δ*spa47*; pUC19 *spa13* with N-terminal FLAG	This study
Δ*spa47*/FLAG_*spa47*	Δ*spa47*; pUC19 *spa47* with N-terminal FLAG	This study

TABLE S2. Primer sequences used in this study*

Primer	Sequence
MxiK_FLAG_For	5'-AGTCTCTAGACAAGAGAAACAAAATATG*GACTACAAGGACGACGATGACAAG*ATAAGAATGGATGGAAT-3'
MxiK_Rev	5'-AGTCGGATCCTCATAGGCATGATGTCTGG-3'
Spa13_FLAG_For	5'-AGTCTCTAGAAGGAGGTTATTGGTG*GACTACAAGGACGACGATGACAAG*AAACAATTAGATAAGG-3'
Spa13_Rev	5'-AGTCGGATCCTTATCTAATGCCATACTTC-3'
Spa47_FLAG_For	5'-AGTCTCTAGATGGGGTCTTATAATCAATG*GACTACAAGGACGACGATGACAAG*AGCTATACAAAATTGCTC-3'
Spa47_Rev	5'-AGTCGGATCCTTATCTAATTGTTTCACCA-3'
Spa33_FLAG_For	5'-AGTCTCTAGAACAGAGTGAAGAAGAATG*GACTACAAGGACGACGATGACAAG*CTAAGAATTAAACATTTT-3'
Spa33_Rev	5'-AGTCGGATCCTTACTCCTTTACCATCCAA-3'
Spa33_For	5'-AGTCTCTAGAACAGAGTGAAGAAGAATGCTAAGAATTAAACATTTT-3'
Spa33_FLAG_Rev	5'-AGTCGGATCCTTA*CTTGTCATCGTCGTCCTTGTAGTC*CTCCTTTACCATCCAAGA-3'

*Underlined capital letters represent restriction endonuclease sites generated to facilitate cloning. Italic capital letters represent FLAG sequence.

REFERENCES

1. **Sansonetti PJ, Kopecko DJ, Formal SB.** 1982. Involvement of a plasmid in the invasive ability of Shigella flexneri. Infection and immunity **35:**852-860.
2. **Kawamoto A, Morimoto YV, Miyata T, Minamino T, Hughes KT, Kato T,**

Namba K. 2013. Common and distinct structural features of Salmonella injectisome and flagellar basal body. Sci Rep **3:**3369.

3. **Jouihri N, Sory MP, Page AL, Gounon P, Parsot C, Allaoui A.** 2003. MxiK and MxiN interact with the Spa47 ATPase and are required for transit of the needle components MxiH and MxiI, but not of Ipa proteins, through the type III secretion apparatus of Shigella flexneri. Mol Microbiol **49:**755-767.
4. **Schuch R, Maurelli AT.** 2001. Spa33, a cell surface-associated subunit of the Mxi-Spa type III secretory pathway of Shigella flexneri, regulates Ipa protein traffic. Infect Immun **69:**2180-2189.
5. **Cherradi Y, Hachani A, Allaoui A.** 2013. Spa13 of Shigella flexneri has a dual role: chaperone escort and export gate-activator switch of the type III secretion system. Microbiology doi:10.1099/mic.0.071712-0.

SUPPLEMENTARY FIGURE LEGENDS

Supplementary Figure S1. Masks for alignment and classification. (a) *Top*, tight mask for alignment. *Bottom,* the mask layered on averaged vT3SS. **(b)** *Top*, cytoplasmic mask for classification. *Bottom*: the mask layered on averaged vT3SS.

Supplementary Figure S2. Gold-standard FSC curves of NC and HBB in *Shigella flexneri/Salmonella* Typhimurim*.* *Top*, two FSC curves show resolution of NC in *Shigella* at 0.143 criterium with C1 (*red*) and C6 symmetry (*blue*). *Middle*, two FSC curves shows resolution of NC in *Salmonella* at 0.143 criterium with C1 (*red*) and C6 symmetry (*blue*) *Bottom*, two FSC curves shows resolution of HBB in *Salmonella* at 0.143 criteria with C1 (*red*) and C6 symmetry (*blue*).

Supplementary Figure S3. Subtomogram averaging and classification of

Hook Basal Body (HBB) of *Salmonella* with C1 and C6 symmetry. (a, b) Central and cross sections of original subtomogram average with C1 and C6 symmetry **(a)** and three classes **(b)** *Below,* each corresponding cross section at height indicated by the dashed yellow line. The number of particles for each class is given on the left of each image. **(c)** Side and bottom views of 3D surface rendering of HBB. Scale bar indicates 10 nm.

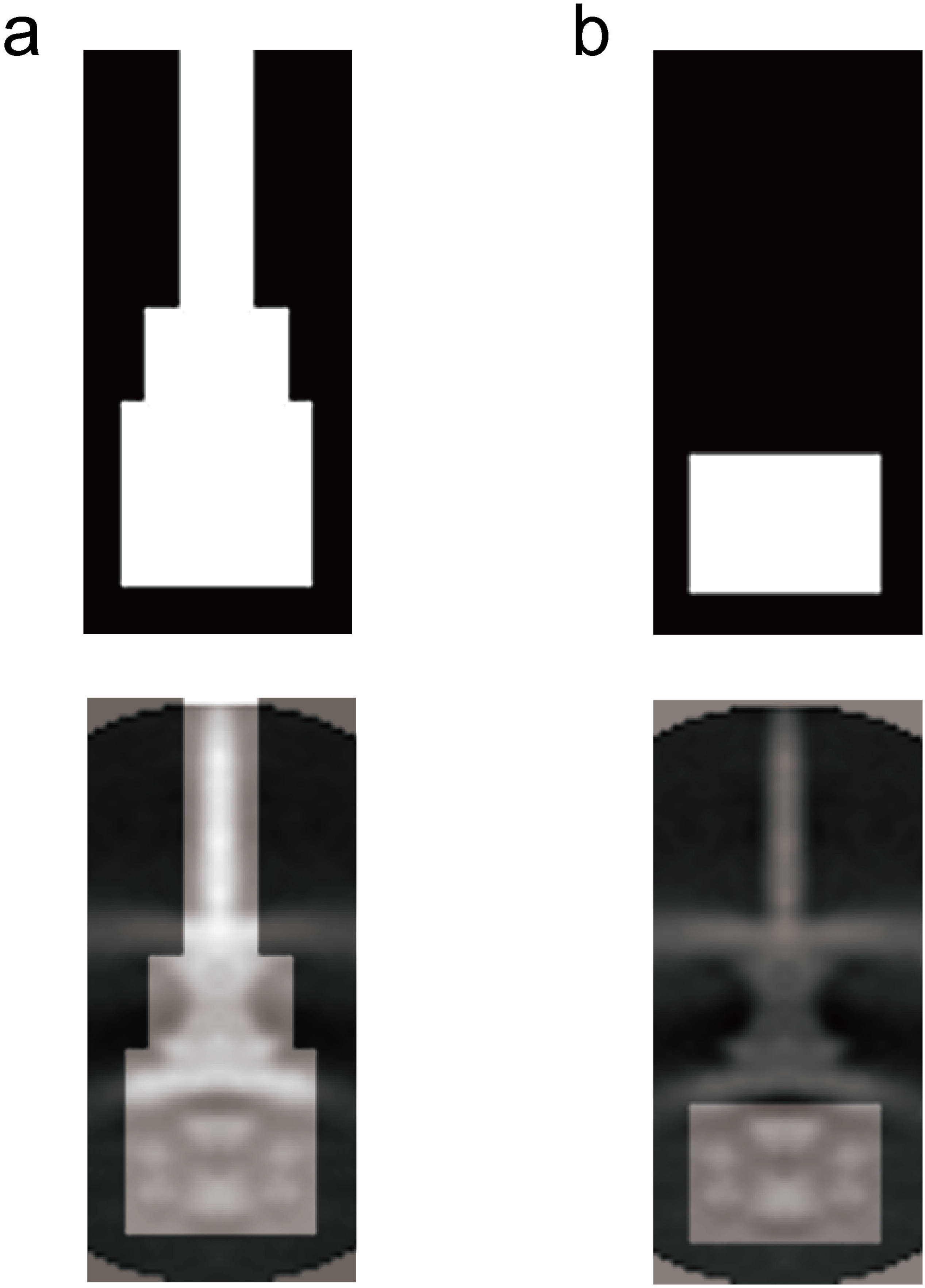

Figure S1

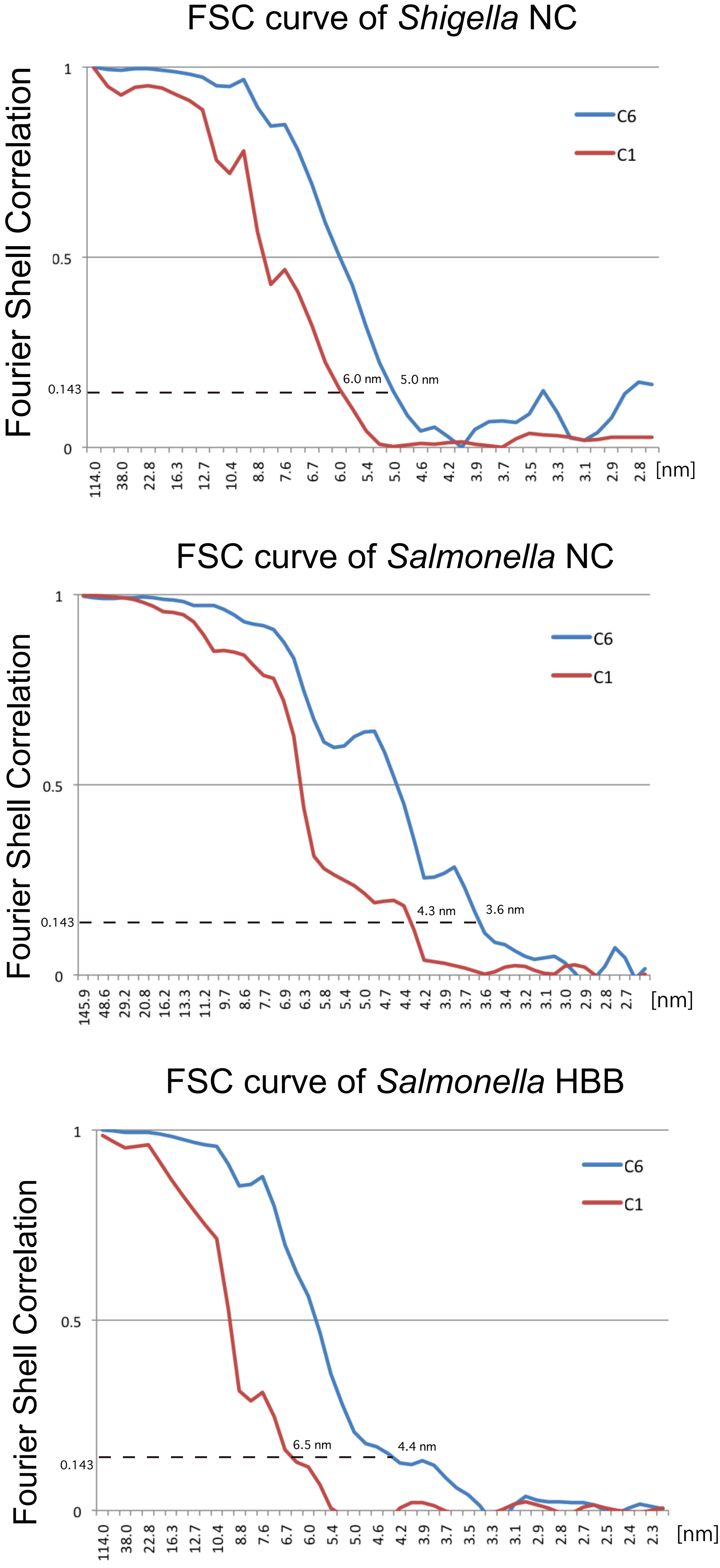

FSC curve of *Shigella* NC
Fourier Shell Correlation
1
0.5
0.143
0
C6
C1
6.0 nm
5.0 nm
114.0 38.0 22.8 16.3 12.7 10.4 8.8 7.6 6.7 6.0 5.4 5.0 4.6 4.2 3.9 3.7 3.5 3.3 3.1 2.9 2.8
[nm]
FSC curve of *Salmonella* NC
Fourier Shell Correlation
1
0.5
0.143
0
C6
C1
4.3 nm
3.6 nm
145.9 48.6 29.2 20.8 16.2 13.3 11.2 9.7 8.6 7.7 6.9 6.3 5.8 5.4 5.0 4.7 4.4 4.2 3.9 3.7 3.6 3.4 3.2 3.1 3.0 2.9 2.8 2.7
[nm]
FSC curve of *Salmonella* HBB
Fourier Shell Correlation
1
0.5
0.143
0
C6
C1
6.5 nm
4.4 nm
114.0 38.0 22.8 16.3 12.7 10.4 8.8 7.6 6.7 6.0 5.4 5.0 4.6 4.2 3.9 3.7 3.5 3.3 3.1 2.9 2.8 2.7 2.5 2.4 2.3
[nm]

Figure S2

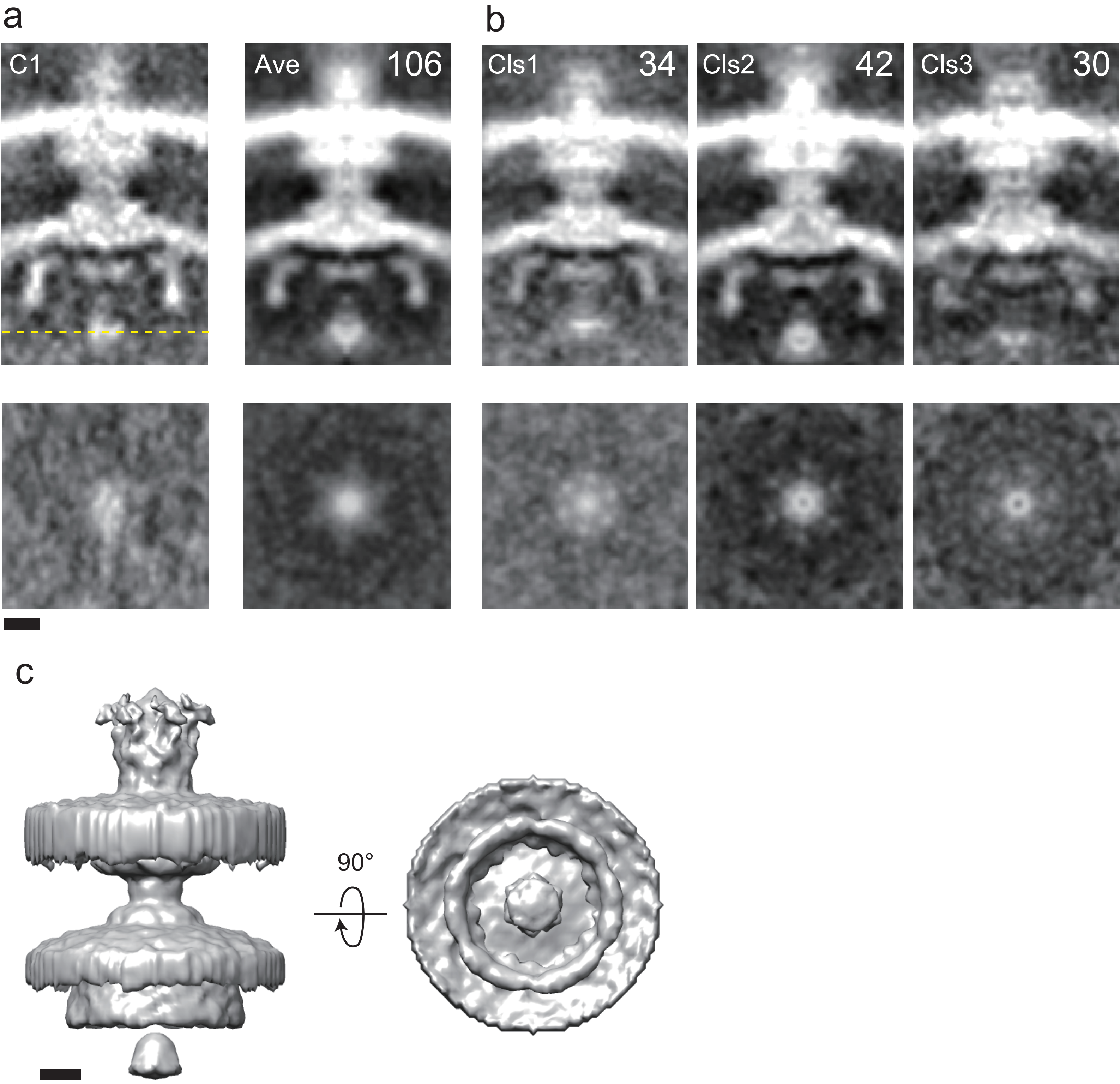

Figure S3
